# News and Notes

**Published:** 1904-09

**Authors:** 


### *NEWS AND NOTES.*

There were 44,783 deaths from plague in India during the week ending April 30.

The fourth Pan-American Medical Congress will meet in Panama the latter part of December.

The National Association for the Study and Prevention of Tuberculosis was organized June 6, at Atlantic City.

The International Congress of Physiologists will hold its next meeting at Brussels this year from August 30 to September 3.

The next meeting of the International Congress for the Study of Tuberculosis will be held in Paris in 1905, from October 2 to 7.

Among the members of the medical profession in foreign countries who have recently died are Dr. Camille Miot, of Paris, one of the pioneers of otology in France, and author of numerous writings on subjects pertaining to that department of medical practice, aged sixty-six; Dr. Francois Jouon, professor of anatomy in the Medical School of Nantes; and Dr. C. Rouget, emeritus professor of physiology in the University of Montpellier.

At the closing of the American Surgical Association, June 17, the following officers were elected for the year 1904–1905: President, George Ben Johnson, Richmond, Va.; Vice-President, Emmett Rixford, San Francisco; secretary, Dudley T. Allen, Cleveland; Recorder, Richard T. Hart, Philadelphia; treasurer, R. S.

Fowler, Brooklyn. San Francisco was chosen as the place of meeting for 1905.

---

AT a recent meeting of the Red Cross Society in Washington, the trouble which has divided the members of the society into two factions was, it is hoped, satisfactorily adjusted, says the *Medical Record*. All the trustees and other officers, with the exception of three, resigned, and a new set was elected. Ex-Surgeon-General W. K. Van Ruypen of the navy was made president, and Dr. Walter Wyman, Surgeon-General of the Public Health and Marine Hospital Service, vice-president. The appointment of a special committee was authorized to adopt resolutions in recognition of the services of Clara Barton, the former president of the society. A resolution complimentary to Miss Barton was offered at the meeting, but it was ruled out of order.

---

ACCORDING to the Bulletin of the New York State Department of Health, 13,700 deaths occurred during the month of April. The Bulletin states that this month has generally the uniform mortality of about 10,500, and is seldom subject to fluctuation. However, the number of deaths for this April is within 300 deaths of April, 1891, and within 600 of that of the month preceding, so that it stands the third highest on the record of monthly death-rates. It is further exceptional in that it is sequent to a series of months of excessive mortality. In the four months of this year there have been 53,000 deaths in this State, against 44,500 for the same months in 1903, and 43,300 in 1902. At this rate for the year there would be 160,000 deaths against 130,000, or against an average yearly mortality of recent past years of from 125,000 to 130,000. The chief increase in mortality is in diseases of the respiratory system, and especially from pneumonia, from which there were 2,500 more deaths than in the four months of the preceding two years. The increase has been general throughout the State.

